# *Coccidioides immitis* Identified in Soil Outside of Its Known Range — Washington, 2013

**Published:** 2014-05-23

**Authors:** Nicola Marsden-Haug, Heather Hill, Anastasia P. Litvintseva, David M. Engelthaler, Elizabeth M. Driebe, Chandler C. Roe, Cindy Ralston, Steven Hurst, Marcia Goldoft, Lalitha Gade, Ron Wohrle, George R. Thompson, Mary E. Brandt, Tom Chiller

**Affiliations:** 1 Washington State Department of Health; 2 Benton-Franklin Health District, Kennewick, Washington; 3 Division of Foodborne, Waterborne, and Environmental Diseases, National Center for Emerging and Zoonotic Infectious Diseases, CDC; 4 Translational Genomics Research Institute, Flagstaff, Arizona; 5 Coccidioidomycosis Serology Laboratory, University of California–Davis

Coccidioidomycosis (“valley fever”) is caused by inhaling spores of the soil-dwelling fungi *Coccidioides immitis* or *Coccidioides posadasii*. Most infections are subclinical. When clinical manifestations do occur (typically 1–4 weeks after exposure), they are similar to those associated with influenza or community-acquired pneumonia. Disseminated disease is rare. Residual pulmonary nodules can lead to chronic lung disease. Fluconazole or other triazoles often are used for treatment, but mild cases often resolve without specific therapy. A total of 17,802 cases were reported in the United States in 2012.

Coccidioidomycosis is endemic to the hot, arid regions of the southwestern United States and Central and South America; Washington state is far north of its recognized range. However, three acute coccidioidomycosis cases among residents of south central Washington reported during 2010–2011 were suspicious for local acquisition; none of the three patients had traveled within 22 months of illness onset to an area where coccidioidomycosis is known to be endemic (1).

In November 2010, during the investigation of the Washington cases, soil was collected from two locations in Benton County, Washington. Although no reliable test for identifying *Coccidioides* in soil existed at that time, environmental testing methods were being studied by CDC and its partners. Sampling sites on public lands were identified by interviewing two patients; one site was a dirt track used for all-terrain vehicle riding and the other was near a residential complex. Soil samples were obtained from locations where patients described falling or playing in the dirt and from nearby rodent burrows and snake holes.

Soil samples were refrigerated at the Washington State Public Health Laboratories until August 2013, when they were sent to CDC’s Mycotic Diseases Laboratory. A novel real-time polymerase chain reaction assay developed by the Translational Genomics Research Institute was used to detect *Coccidioides* DNA in six of 22 soil samples. Viable *C. immitis* was isolated from four of the six soil samples using a modified yeast extract medium. Sequencing of rDNA and three other genes confirmed the isolates as *C. immitis*. The environmental isolate genotypes were identical to a clinical isolate from one patient by whole genome sequencing. This is new direct evidence that the infections were acquired in Washington and that *C. immitis* exists in this environment clearly outside the recognized endemic area.

Health-care providers should be aware that *C. immitis* is present in south central Washington, and should consider the diagnosis in patients with clinically compatible illness who reside or have traveled in this area. Furthermore, health-care providers in surrounding regional areas should consider testing for coccidioidomycosis if clinically warranted and exposures like those described exist. Similarly, veterinarians should be aware of the possibility of *Coccidioides* infection outside its recognized range. Further work to understand the geographic range of this disease is underway. Coccidioidomycosis is a nationally notifiable disease; reporting to public health authorities helps describe the occurrence of cases in new areas.
